# Multinational comparison of the detection of extended-spectrum beta-lactamase genes in healthy resident feces

**DOI:** 10.1128/spectrum.02920-24

**Published:** 2025-04-30

**Authors:** Yoshimasa Yamamoto, Hoa Thi Thanh Hoang, Yen Hai Le, Cornelia Appiah-Kwarteng, Diep Thi Khong, Thang Nam Nguyen, Manuel Calvopina, Carlos Bastidas-Caldes, Nobuyuki Tetsuka, Masaki Anraku, Mayumi Yamamoto

**Affiliations:** 1 The United Graduate School of Drug Discovery and Medical Information Sciences, Gifu University 12785, Gifu, Japan; 2 School of Veterinary Medicine, University of Ghana 58835, Accra, Ghana; 3 Center for Medical and Pharmaceutical Research and Service, Thai Binh University of Medicine and Pharmacy 71382, Thai Binh, Vietnam; 4 One Health Research Group, Universidad De Las America 149235, Quito, Ecuador; 5 Department of Infection Control, School of Medicine, Gifu University 12785, Gifu, Japan; 6 Department of Microbiology, Osaka Institute of Public Health 91397 , Osaka, Japan; 7 Health Administration Center, Gifu University 12785, Gifu, Japan; JMI Laboratories, North Liberty, Iowa, USA

**Keywords:** extended-spectrum beta-lactamase genes, feces, residents, real-time PCR, multinational

## Abstract

The spread of antimicrobial-resistant bacteria, especially in developing countries, is a critical healthcare issue. Among these, extended-spectrum beta-lactamase (ESBL)-producing bacteria are particularly concerning due to their resistance to third- and fourth-generation cephalosporins. Traditional methods for assessing bacterial resistance involve culturing bacteria on selective media from fecal samples, which may lead to selection bias. Alternatively, real-time PCR allows for detecting resistance genes directly from fecal DNA, providing a broader view of resistant bacteria. In this study, we evaluated the utility of a real-time PCR assay targeting ESBL-producing genes as a comprehensive detection method for ESBL-producing resistant bacteria in fecal samples. Additionally, we conducted a multinational comparative analysis of the colonization status of residents using this approach. The study analyzed ESBL genes in fecal samples from 161 residents in four countries: Ecuador, Ghana, Vietnam, and Japan. Samples from Ecuador, Ghana, and Vietnam, where ESBL carriage was notably high, revealed gene variations by country, with *bla*TEM genes being most common except in Ghana, where *bla*SHV genes predominated. These variations suggest that different bacterial hosts carry ESBL genes across countries. Quantitative PCR results further highlight that *bla*TEM is the most abundant ESBL gene. Although gene presence does not confirm antibiotic resistance, these findings underline significant ESBL carriage in low- and middle-income countries. The study emphasizes that gene detection in fecal samples is valuable for understanding resistant bacteria spread in communities.

**IMPORTANCE:**

The rise of antimicrobial-resistant bacteria, particularly extended-spectrum beta-lactamase (ESBL)-producing strains, poses a serious threat to healthcare in developing countries. This study utilized real-time PCR to detect ESBL genes directly from fecal DNA of 161 participants across four countries, offering a comprehensive analysis without the biases of traditional culture-based methods. High ESBL gene carriage rates were found in Ecuador, Ghana, and Vietnam, with regional differences in gene prevalence: *bla*TEM dominated in most countries, while *bla*SHV was most frequent in Ghana. These results highlight the widespread community-level dissemination of ESBL genes in low- and middle-income countries, underscoring the importance of using gene detection as a tool for assessing the spread of resistant bacteria.

## OBSERVATION

The emergence and spread of antimicrobial-resistant bacteria pose a serious threat to healthcare. This threat is particularly severe in developing countries, where appropriate medical resources are often lacking. Among the resistant bacteria, extended-spectrum beta-lactamase (ESBL)-producing bacteria are of particular concern as they are resistant to commonly used third- and fourth-generation cephalosporins and penicillins, making their spread a significant issue (1).

Assessment of carriage of resistant bacteria is typically performed by isolating and culturing bacteria from fecal samples using selective media, followed by analysis of their resistance profiles. However, this method can introduce a selection bias owing to the use of selective media, which may not accurately reflect the overall prevalence of resistant bacteria within the microbial flora. Alternatively, the detection of resistance genes in DNA extracted from fecal samples using real-time PCR enables a more quantitative and comprehensive evaluation independent of the bacterial species present. However, the detection of resistance genes does not always correlate with that of resistant bacteria.

Many studies have used bacterial isolation and culture methods to investigate the prevalence and characteristics of ESBL-producing bacteria in healthy residents. For example, a study reported that 5.8% of Swiss residents harbored ESBL-producing *Escherichia coli*, with 97% of the ESBL genes identified as the CTX-M type (2). Similarly, in our study, 51%–71% of residents in Asian countries carried ESBL-producing bacteria, with 92%–97% of these isolates harboring CTX-M genes (3). Despite country-specific differences in ESBL carriage rates, CTX-M remains the predominant ESBL type (4, 5).

Interestingly, a study using PCR to evaluate fecal DNA from Indonesian residents found that 76% of schoolchildren harbored ESBL genes, with 92% of the TEM type and only 4.3% of the CTX-M type (6). Whether these differences are attributable to geographic factors or to the use of different detection methods (culture-based vs molecular detection) remains uncertain.

In this study, we used real-time PCR to detect and quantify ESBL genes in fecal samples from residents and conducted a comparative analysis across multiple countries, including Ecuador, Ghana, Vietnam, and Japan. To the best of our knowledge, this is the first report to provide a comprehensive evaluation of ESBL genes in fecal samples from multiple countries.

Fecal samples were collected from 161 participants across four countries between August 2023 and April 2024. The details of the countries, cities, and participants are listed in Table 1. One fecal sample was collected from each participant. The collected fecal samples were transported to the laboratory in a cooler box containing an ice gel pack. In the laboratory, 1 g of feces was diluted in 9 mL of sterile distilled water. One milliliter of the resulting suspension was centrifuged at 1,500 rpm (211 × *g*) for 1 min, and the supernatant was collected. The supernatant was centrifuged at 15,000 rpm (15,871 × *g*) for 5 min to obtain a pellet. DNA was extracted from the resulting pellets using the Kaneka Easy DNA Extraction Kit version 2 (Kaneka, Tokyo, Japan), following the manufacturer’s protocol. Approximately 100 µL of DNA extract was obtained from each sample.

**TABLE 1:** Participants’ characteristics

Country	Area	City	Number of participants	Age		Sex
				Median	Range	Male (%)
Ecuador	Pacific coastal	Sto Domingo	55	44	11–84	27
Ghana	Municipal capital	Ejisu	27	41	11–85	29.6
Vietnam	Red River Delta	Thai Binh	50	41	3–93	46
Japan	Central Japan	Gifu	29	20	18–54	17

ESBL genes were detected in the extracted DNA using a previously published probe-based real-time PCR method (7). To detect *bla*CTX-M genes, the *bla*CTX-M-1 group and other groups (CTX-M-U) were assessed separately for technical reasons. The *bla*TEM and *bla*SHV genes were detected using universal primers and probes covering all subtypes (Table S1). The detection sensitivity for resistance genes was 10^3^ copies per gram of feces.

As shown in Table 2, fecal samples from residents of Ecuador, Ghana, and Vietnam, which are low- and middle-income countries, had significantly higher positive rates of ESBL genes than fecal samples from Japanese residents (chi-squared test, *P* <0.05). This indicated a higher prevalence of ESBL-producing bacterial carriers in these countries. Notably, in Ghana, nearly all residents harbored some ESBL genes, suggesting widespread dissemination of ESBL-producing bacteria in communities. When examining the positivity rates for each ESBL gene, including *bla*CTX-M, *bla*TEM, and *bla*SHV, the predominant ESBL genes varied by country. However, excluding Ghana, where *bla*SHV was detected in almost all residents tested, *bla*TEM was the most commonly detected ESBL gene. More than half of the residents in Ecuador, Ghana, and Vietnam carried the *bla*TEM gene, followed by *bla*SHV and *bla*CTX-M. Considering that *bla*CTX-M and *bla*TEM are primarily carried by members of *Enterobacteriaceae*, including *Escherichia coli*, whereas *bla*SHV is common in *Klebsiella pneumoniae* besides *E. coli* (8), the differences in the ESBL gene spectrum between countries may reflect the differences in the bacterial species carrying these genes. However, the details of this process remain unclear.

**TABLE 2:** Detection of antimicrobial resistance (AMR) genes in resident fecal samples

Country	Sampling date	No. of samples tested		No. of samples positive for AMR genes		
			No. of samples positive for ESBL genes	ESBL genes		
				blaCTX-M^*a*^	blaTEM	blaSHV
Ecuador	23 Aug	55	37 (67.2%)	15 (27.3%)	34 (61.8%)	30 (54.5%)
Ghana	23 Dec	27	26 (96.3%)	11 (40.7%)	22 (81.5%)	26 (96.3%)
Vietnam	24 Mar	50	37 (74%)	17 (34%)	31 (62%)	15 (30%)
Japan	24 Apr	29	12 (41.3%)	2 (6.9%)	7 (24%)	5 (17.2%)

^*a*^ The number of positive samples in either the blaCTX-M-1 group or the other group (CTX-M-U) is shown.

**Fig 1:**
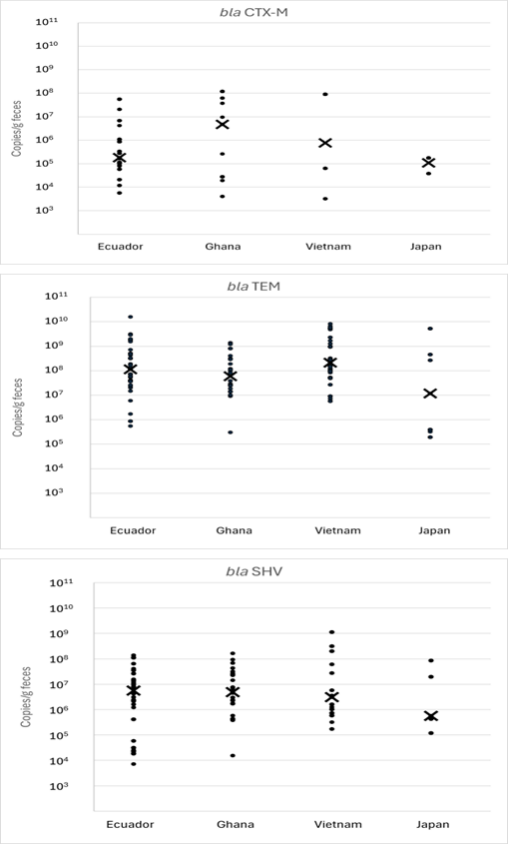
Quantitative levels of ESBL genes in resident feces. X in the figure indicates the median level of positive samples.

The levels of ESBL genes in the fecal samples are shown in Fig. 1. The concentration ranges of all detected genes in the fecal samples were broad, revealing significant variation in the carriage levels of these gene-harboring bacteria among residents. Notably, the quantitative level of the *bla*TEM gene was higher than that of the other genes examined, indicating that *bla*TEM is the predominant ESBL gene in the populations studied, not only in terms of detection rate but also in terms of quantitative levels.

In this study, we used ESBL genes as markers of ESBL-producing resistant bacteria that harbor these genes. However, the presence of multiple resistance genes in a bacterium or the presence of resistance genes does not necessarily indicate resistance to the antibiotics. Therefore, the results obtained do not directly reflect the carriage status of ESBL-producing resistant bacteria, which is a limitation of the present study. Nevertheless, information on the presence and quantitative levels of resistance genes is important for a comprehensive assessment of carriage status among residents. Thus, this study strongly suggests widespread carriage of ESBL-producing bacteria among residents of low- and middle-income countries. In addition, the detection of resistance genes in fecal samples from residents is a useful indicator of community-wide dissemination of resistant bacteria.

## SUPPLEMENTAL MATERIAL

Table S1 spectrum.02920-24-s0001.xlsx Primers and probes used in the study.

ASM does not own the copyrights to Supplemental Material that may be linked to, or accessed through, an article. The authors have granted ASM a non-exclusive, world-wide license to publish the Supplemental Material files. Please contact the corresponding author directly for reuse.
